# Prognostic Nomogram for Postoperative Hypopharyngeal Squamous Cell Carcinoma to Assist Decision Making for Adjuvant Chemotherapy

**DOI:** 10.3390/jcm11195801

**Published:** 2022-09-30

**Authors:** Di Zhang, Lixi Li, Tingyu Wen, Yun Wu, Fei Ma

**Affiliations:** Department of Medical Oncology, National Cancer Center/National Clinical Research Center for Cancer/Cancer Hospital, Chinese Academy of Medical Sciences and Peking Union Medical College, Chaoyang District, Panjiayuan Nan Road 17, Beijing 100021, China

**Keywords:** hypopharyngeal squamous cell carcinoma, lymph node parameters, chemotherapy, prognosis, nomogram model, surveillance, epidemiology, and end results database

## Abstract

We aimed to investigate the effect of lymph node parameters on postoperative hypopharyngeal squamous cell carcinoma (HSCC) and to establish a nomogram to predict its prognosis and assist in adjuvant chemotherapy decisions. A retrospective analysis of postoperative HSCC in the Surveillance, Epidemiology, and End Results database (2004–2019) was performed. Cutoff points for continuous variables were determined by X-tile software. Univariate and multivariate analyses were performed to identify prognostic factors on overall survival (OS), and these variables were used to construct a nomogram. The nomogram’s accuracy was internally validated using concordance index, area under the curve, calibration plot, and decision curve analyses. Furthermore, the value of chemotherapy in each risk subgroup was assessed separately based on individualized scores from the nomogram. In total, 404 patients were eligible for analysis, and the median OS was 39 months. Age, origin, primary site, T stage, number of lymph nodes examined, lymph node ratio, and radiotherapy were identified as prognostic factors for OS and incorporated into the nomogram. In both the training and validation cohorts, favorable performance was exhibited compared with the other stage systems, and patients could be classified into low-, intermediate-, and high-risk subgroups. Chemotherapy significantly improved the OS in the high-risk subgroup, whereas chemotherapy did not confer a survival benefit in the low- or intermediate-risk groups. The lymph node parameter-based nomogram model can better stratify the prognosis of HSCC patients and screen out patients who would benefit from chemotherapy, suggesting that the model could be used as a reference for clinical decision making and to avoid overtreatment.

## 1. Introduction

Hypopharyngeal carcinoma is rare in clinical practice, accounting for 2–6% of head and neck malignancies [1], mainly squamous cell carcinoma [2]. Hypopharyngeal squamous cell carcinoma (HSCC) is mostly located in the piriform fossa, and early diagnosis is difficult due to the occulted location of lesions [3]. There is a rich submucosal lymphatic network in the hypopharynx, which promotes cervical lymph node metastasis at an early stage [4,5]. Moreover, HSCC is characterized by a high degree of malignancy and rapid growth [6,7]. Comprehensive treatment based on surgery is still the first choice for hypopharyngeal cancer [8,9,10]. However, the five-year survival rate of HSCC is 25–35%, and its overall outcome remains non-ideal [11,12]. Therefore, it is essential to pay particular attention to the prognosis problems of HSCC patients.

The American Joint Committee on Cancer (AJCC) tumor–lymph node–metastasis (TNM) staging system is the most commonly used prognostic model for patients with head and neck cancer. Of these, the lymph node stage depends on the number, size, laterality, and extra-nodal extension status of the regional lymph nodes [13], but does not include the burden of lymph node metastasis. It has been reported that the number of examined lymph nodes (ELN) and the number of positive lymph nodes (PLNN) are also closely associated with survival outcome for patients with head and neck cancer [14,15]. In addition, the lymph node ratio (LNR) refers to the proportion of metastatic lymph nodes in the total number of detected lymph nodes [16,17], theoretically providing greater prognostic value [18,19]. A higher LNR may mean a higher possibility of potential regional recurrence, and, thus, has greater significance for the selection of adjuvant therapy [20,21,22]. To sum up, a better postoperative prognosis assessment system for patients with HSCC is conducive to the selection of appropriate patients for more intensive adjuvant therapy and for the further design of clinical trials. 

In this study, we constructed a nomogram model based on lymph node parameters and clinical characteristics to stratify the prognosis of patients with HSCC after surgery. The purpose of this study was to evaluate the role of chemotherapy in different risk stratifications to promote the precision of treatment of HSCC.

## 2. Materials and Methods

### 2.1. Data Collection

Data from this study population were retrieved from the Surveillance, Epidemiology, and End Results (SEER) database between 1 January 2004 and 31 December 2019. Supplementary Figure S1 shows this in more detail. As the data for this study were derived from a public database, there was no need for additional ethical application.

The main inclusion criteria were as follows: (1)The site of the disease was located in the hypopharynx (C12.9–C13.9); (2) the histology was limited to squamous cell neoplasms (8050–8090); (3) first primary malignancy; (4) surgery of the primary tumor site was performed, and surgery methods were limited to pharyngectomy, pharyngectomy with laryngectomy, or removal of contiguous bone tissue, and radical pharyngectomy (code 30–52); (5) ELN ≥ 1; (6) AJCC TNM stage of T1-4N0-3M0. 

Patients were excluded if the following criteria were met: (1) Repeated ID; (2) T0, TX, or NX stage; (3) distant metastasis; (4) PLNN or ELN was unknown; (5) without surgery of the primary site, or the surgery method was local resection or NOS; (6) the radiotherapy method was preoperative and intraoperative radiotherapy.

### 2.2. Variable Definition

Overall survival (OS) was defined as the time from definitive diagnosis to death from any cause or the last follow-up. Disease-specific death (DSS) was calculated as the interval from initial diagnosis to the date of cancer-specific death or the last follow-up. The LNR was defined as the number of positive regional nodes (1988+) divided by the number of regional nodes examined (1988+).

### 2.3. Statistical Analysis

The appropriate threshold was obtained using X-tile software (version 3.6.1; Yale University, New Haven, CT, USA). The OS was analyzed using the Kaplan–Meier method and compared using the Log-rank test. Cox multivariate proportional hazards regression was used to identify the independent factors for OS, and the nomogram model was constructed. The C-index, time-dependent receiver operating characteristic (ROC) curve and corresponding area under curve (AUC), calibration curves, and decision curve analysis (DCA) were used to evaluate the prediction efficiency of the model. The R package used in the analysis mainly included “ggplot2”, “survival”, “survminer”, “rms”, “pROC”, “plotROC”, “survivalROC”, “timeROC”, “dplyr”, “pec”, and “ggDCA”. Statistical analyses were performed using SPSS software (version 23.0; IBM, Armonk, NY, USA) and R software (version 4.1.1; R Foundation Statistical Computing, Vienna, Austria), and figures were produced using GraphPad Prism (version 9.0; GraphPad Software Inc., San Diego, CA, USA). *p* < 0.05 was considered statistically significant.

## 3. Results

### 3.1. General Characteristics and Treatment Patterns

Overall, 404 patients with HSCC who underwent surgery were selected from the SEER database from 2004 to 2019. The patients were randomly divided into a training cohort (*n* = 282) and a validation cohort (*n* = 122) at a ratio of 7:3. The clinical characteristics, histopathologic information, and treatment details are shown in Table 1. There was no significant difference in the general characteristics between the training and validation cohorts (all *p* > 0.05).

In the whole cohort, the predominant patients were male (82.92%) and White (77.48%), and the median age at diagnosis was 62 years. Nearly 60% of primary tumors were located in the pyriform sinus, and the median tumor size was 40 mm. Most patients had lymph node metastasis; the median ELN, PLNN, and LNR were 43, 2, and 0.06, respectively. In addition, 74.76% of the cases were stage III–IV, and over 90% were histological grade II–III. All patients underwent pharyngectomy, and 75% of the patients received postoperative radiotherapy, while nearly half of patients (49.01%) received chemotherapy.

### 3.2. Identification of Optimal Cutoff Points

The optimal cutoff points of age at diagnosis, ELN, PLNN, and LNR, were obtained using X-tile software, and the corresponding Kaplan–Meier curves showed significant statistical differences among each subgroup (Supplementary Figure S2). The results showed that the cutoff point for age at diagnosis was 71 years old (*n* = 233 vs. 49, *χ*^2^ = 9.218, relative risk = 1.00/1.17, *p* < 0.05). The optimal cutoff points for ELN were 36 and 72 and for PLNN were 1 and 6. Moreover, the cutoff points for the LNR were 0.03 and 0.23 (*n* = 99 vs. 143 vs. 40, *χ*^2^ = 19.672, relative risk = 1.00/1.34/1.55, all *p* < 0.05).

### 3.3. Survival Analysis in the Entire Cohort Population

The median follow-up time of the entire population was 93 months. Among these patients, 189 patients died from HSCC, and 77 patients died from other causes, including diseases of the heart, lung, and the bronchus, and miscellaneous malignant cancer. The median OS of the patients was 39 months, and the median DSS was 57 months. The overall one-, three-, and five-year OS rates of the HSCC patients were 77.99%, 52.88%, and 39.43%, respectively. The DSS rate at one, three, and five years were 79.81%, 59.37%, and 48.86%, respectively.

### 3.4. Univariate and Multivariate Analysis in the Training Cohort

As presented in Table 2, univariate and multivariate analyses were conducted in the training cohort to identify the prognostic factors for OS. The results of the univariate analysis indicated that age, primary site, T stage, N stage, tumor size, ELN, PLNN, LNR, and postoperative radiotherapy were significantly associated with OS in patients with HNSCC after surgery (all *p* < 0.05). In the multivariate Cox regression model, age, origin, primary site, T stage, ELN, LNR, and radiotherapy were independent prognostic factors for OS in the patients with HSCC after resection (all *p* < 0.05). Compared with those with an LNR <0.03, the risk of death was significantly increased in patients with an LNR ≥ 0.03 (HR = 2.721, 95% CI = 1.492–4.963, *p* < 0.001) and an LNR ≥ 0.23 (HR = 3.776, 95% CI = 1.713–8.324, *p* < 0.001). Moreover, patients in the ELN ≥ 73 subgroup had worse survival outcomes than those in the ELN < 37 subgroup (HR = 1.947, 95% CI = 1.153–3.287, *p* = 0.013). However, no significant differences were observed between groups in the year of diagnosis, race, sex, married, grade, N stage, tumor size, LNN, or surgery method after adjusting the entire variables.

### 3.5. Generation of a Nomogram Model

**Figure 1:**
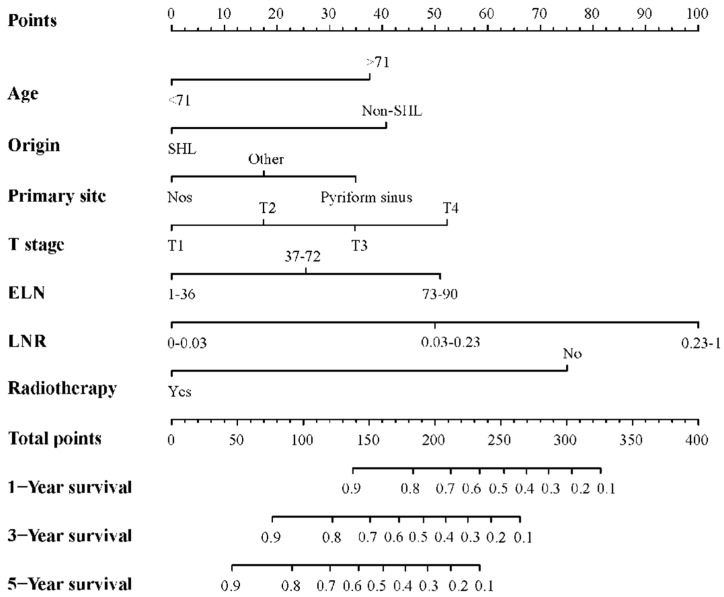
Nomogram model of OS in patients with HSCC after surgery. Abbreviation: OS, overall survival; HSCC, hypopharyngeal squamous cell carcinoma; SHL, Spanish–Hispanic–Latino; Non-SHL, Non-Spanish–Hispanic–Latino; ELN, examined lymph nodes; LNR, lymph node ratio.

Subsequently, a nomogram model to predict OS in HSCC patients after surgery was established, based on all of the independent prognostic factors influencing OS identified in the above multivariate Cox analysis. Each variable was scored using the established model, and the OS probabilities of one, three, and five years for each patient could be estimated by calculating the total score and drawing a plummet line. The details are shown in Figure 1.

### 3.6. Nomogram Validation

The prognostic model was thoroughly evaluated and internally validated for discrimination and calibration. In the training cohort, the C-index of our nomogram model was 0.716, which was better than the traditional AJCC TNM stage system (0.716 vs. 0.558) and the SEER combined stage system (0.716 vs. 0.532). The C-index in the validation cohort was also higher than that of the TNM and SEER stage systems, indicating that the identification ability of our nomogram model is acceptable.

**Figure 2:**
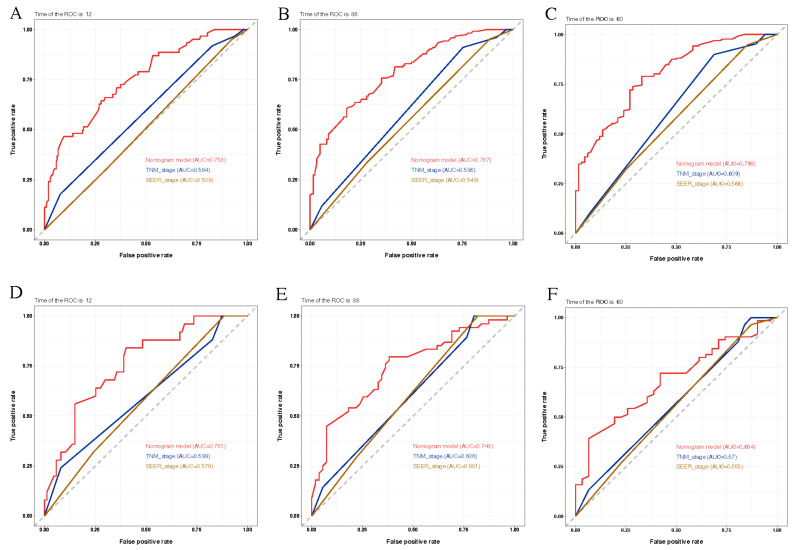
The ROC curves for the one-, three-, and five-year OS rates. (**A**–**C**) Comparison of the ROC curves of the nomogram, TNM and SEER stage for one-year (**A**), three-year (**B**), and five-year (**C**), OS rate in the training cohort. (**D**–**F**) Comparison of the ROC curves of the nomogram and TNM stage for one-year (**D**), three-year (**E**), and five-year (**F**), OS rate in the validation cohort. Abbreviation: ROC, receiver operator characteristic; OS, overall survival; SEER, Surveillance, Epidemiology, and End Results.

Our results further showed that the AUC values of the nomogram model exhibited favorable sensitivity and specificity. In the training cohort, our nomogram model outperformed the AJCC TNM and SEER stage systems in terms of the AUC values for the one-, three-, and five-year OS rates (one-year: 0.753 vs. 0.784 vs. 0.509; three-year: 0.787 vs. 0.596 vs. 0.549; five-year: 0.798 vs. 0.609 vs. 0.566). Similar results were obtained in the validation cohort. More details are shown in Figure 2.

**Figure 3:**
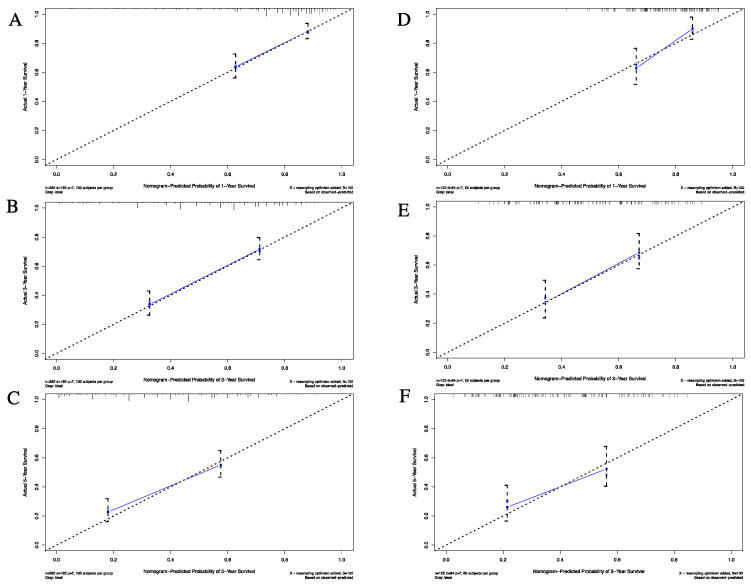
Calibration plot for the one-, three-, and five-year overall survival rates. (**A**)–(**C**) Calibration plot for the one-year (**A**), three-year (**B**), and five-year (**C**), overall survival rates in the training cohort. (**D**–**F**) Calibration plot for the one-year (**D**), three-year (**E**), and five-year (**F**), overall survival rate in the validation cohort.

**Figure 4:**
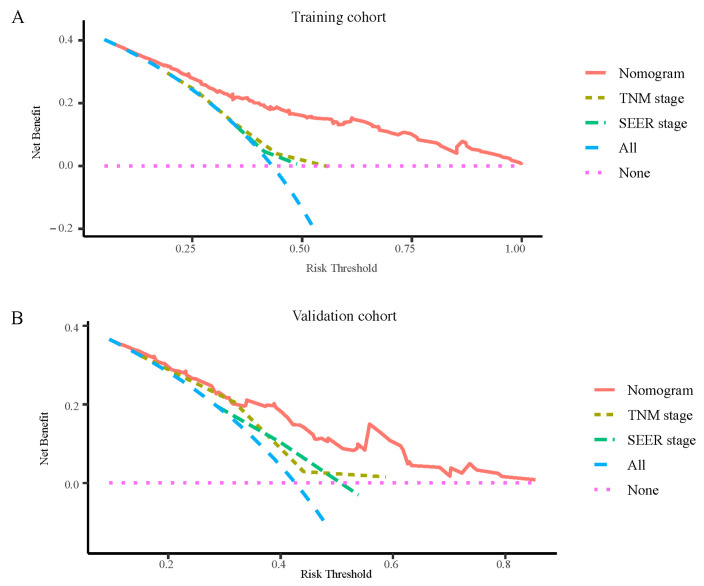
DCA curves of the nomogram, TNM stage, and SEER stage. (**A**) DCA curves of the nomogram, TNM stage, and SEER stage in the training cohort. (**B**) DCA curves of the nomogram, TNM stage, and SEER stage in the validation cohort. Abbreviation: DCA, decision curve analysis; SEER, Surveillance, Epidemiology, and End Results.

In addition, calibration plots with a slope close to 45° are presented in Figure 3. The calibration plots for the one-, three-, and five-year OS predictions showed satisfactory agreement between the actual and predicted clinical outcomes, indicating that the accuracy of our nomogram was satisfactory in both the training and validation cohorts. Furthermore, as shown by the DCA curves, our nomogram demonstrated better net clinical benefit compared with the other models, further validating its superior predictive power and accuracy (Figure 4).

### 3.7. Clinical Value of Nomogram Risk Stratification

**Figure 5:**
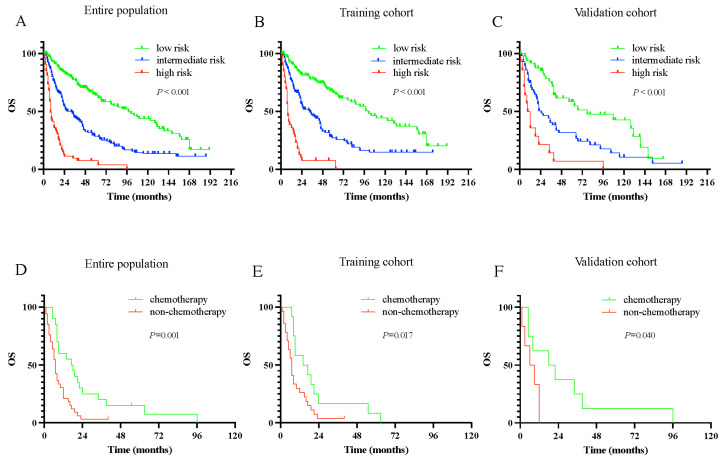
The overall survival of the patients in different nomogram-stratified risk group and the impact of chemotherapy within the various risk subgroups. (**A**–**C**) Overall survival in different nomogram-stratified risk group in the entire population (**A**), the training cohort (**B**), and the validation cohort (**C**). (**D**–**F**) Comparison of chemotherapy and non-chemotherapy within the various risk subgroups in entire population (**D**), the training cohort (**E**), and the validation cohort (**F**).

On the basis of the training cohort, the total score for each patient was calculated according to the nomogram model, and its optimal cutoff points were determined to be 168 and 229 using X-tile software (*χ*^2^ = 110.169, relative risk = 1.00/1.53/2.00, *p* < 0.01). Correspondingly, patients were divided into three new prognostic risk cohorts, namely, low risk (≤168), intermediate risk (168–229), and high risk (>229). Notably, the OS of the patients with HSCC decreased significantly with increasing risk classification. Significant differences in the Kaplan–Meier curves were observed between the different risk subgroups in the entire, training, and validating cohorts (Figure 5). 

Furthermore, the survival benefit of chemotherapy in each risk subgroup was investigated based on our novel classification system. For the entire population cohort, in the high-risk subgroup, the median OS was 17 months for those patients who received chemotherapy and only seven months for those who did not (*p* = 0.001). However, in the low- and intermediate-risk subgroups, there was no statistical difference in survival outcomes between those patients who received chemotherapy and those who did not (all *p* > 0.05). Moreover, for the training cohort, chemotherapy significantly improved the OS in the high-risk subgroup (*p* = 0.017). A similar trend was also illustrated in the high-risk subgroup of the validation cohort, with patients receiving chemotherapy having longer OS than those not receiving chemotherapy (*p* = 0.040).

## 4. Discussion

In this population-based study, a satisfactory nomogram model in postoperative HSCC was established. Compared with other stage systems, our nomogram was more accurate in predicting prognosis and could significantly stratify patients into different risk subgroups. More importantly, we further shed light on the clinical value of chemotherapy based on this novel classification system, providing a reference for clinical practice.

In our study, the majority of patients presented at an advanced stage with lymph node metastases, suggesting that HSCC is an aggressive head and neck malignancy. Even if the patients underwent surgery, the five-year OS was only 39.43%; thus, it is necessary to propose models to monitor recurrence and predict prognosis. Accumulating evidence suggests that the LNR is superior to the N stage or PLNN as a prognostic factor [16,23,24]. The LNR is the ratio of PLNN to ELN and is a better indicator of lymph node burden; thus, it has important clinical significance [25,26,27]. In our study, those patients with an LNR ≥0.03 exhibited significantly decreased OS than those with an LNR < 0.03, implying that a certain number of lymph nodes should be removed in HSCC, and this number is affected by the PLNN. A high LNR may be associated with inadequate neck resection, insufficient pathological examination, or later stage, suggesting a greater likelihood of local recurrence and a greater benefit from adjuvant therapy [13,28,29].

In addition to the LNR, other prognostic factors including age, origin, primary site, T stage, ELN, and radiotherapy were also applied to the model construction. Our nomogram was evaluated with multiple identification and calibration methods, all of which showed good performance. Meanwhile, the TNM and SEER stages were used as a control, further affirming the efficacy of our nomogram model. In previous reports, metastatic patients were also included in the analysis [30,31], but it is worth noting that local therapy should be performed in selected patients, and metastatic patients themselves have worse prognosis, so simply incorporating these variables into the nomogram is inappropriate. However, our nomogram focused only on patients with non-metastatic HSCC undergoing pharyngectomy. For example, Tian et al. [32] developed a nomogram model using the SEER database (2010 and 2016) to predict survival in patients with HSCC, including stage IVC. The one-, three-, and five-year AUC values of their model were 0.748, 0.741, 0.731, respectively. Distinguishingly, we aimed to establish and evaluate a nomogram for postoperative patients, and further analyzed the ability of the model in guiding individual postoperative treatment. Our model had higher AUC values than the traditional stage system and previous report [32] (one-year: 0.753 vs. 0.748; three-year: 0.787 vs. 0.741; five-year: 0.798 vs. 0.731), and it could serve as a stratification indication for adjuvant chemotherapy. Overall, the current study selected the latest SEER data, constructed a nomogram based on lymph node parameters, and proposed a novel risk classification strategy, which has more guiding and far-reaching significance for current clinical management. Patients stratified into the low-risk subgroup had relatively ideal prognosis. In clinical practice, more attention should be given to those in the intermediate- or high-risk subgroups.

Platinum-based concurrent chemoradiotherapy is recommended for HSCC patients with adverse postoperative risk factors, especially those with positive margins or extra-nodal extension [33,34,35]. High-dose cisplatin (100 mg/m^2^ every 3 weeks for up to three cycles) plus RT (60 Gy administered in 2.0 Gy fractions over 7 weeks) is the commonly used chemoradiotherapy [36]. When carboplatin-fluorouracil are used, the recommended regimen is standard fractionation plus three cycles of chemotherapy [37]. Other fractionation sizes, multi agent chemotherapy, other dosing regimens of cisplatin, or altered fractionation with chemotherapy have also been shown to be effective, but there is no consensus on the optimal strategy. Heng et al. [38] developed a nomogram to predict postoperative survival in HSCC patients and proposed that patients with high risk factors could benefit from postoperative radiotherapy or chemoradiotherapy. In Hochfelder et al.’s study, postoperative chemoradiotherapy was associated with significantly prolonged OS and DSS for patients with HSCC [39]. In addition, chemotherapy was also found to significantly reduce mortality in HSCC patients, especially in patients with T3–4 stage [32]. However, some studies have pointed out that chemotherapy does not improve the postoperative prognosis of HSCC patients, which may be related to the toxicity of chemotherapy, which can offset the effect of active treatment [40]. It also has been reported that the five-year OS rate of surgery plus chemoradiotherapy is comparable to that of surgery and postoperative radiotherapy [41]. Therefore, whether HSCC patients can achieve long-term survival benefits from postoperative chemotherapy remains controversial. Moreover, most clinical trials analyze concurrent chemoradiotherapy as a whole, and there are no models to guide the decision making of adjuvant chemotherapy.

In this study, we explored the role of chemotherapy in each subgroup according to risk stratification. Our data demonstrated that chemotherapy was only associated with improved survival outcomes in patients in the high-risk group, but not in patients in the low- and intermediate-risk groups, suggesting that for postoperative HSCC patients, treatment-related toxicity and survival benefit should be considered comprehensively to obtain a more reasonable and individualized treatment strategy. For low-risk patients, appropriate reduction of treatment intensity can be considered, while for high-risk patients, chemotherapy may bring survival benefits if well tolerated by patients.

This study has certain limitations. Some clinicopathological information was not available in the SEER database, such as comorbidities, surgical margins, nerve invasion, and vascular tumor thrombus, which may affect the comprehensiveness of this nomogram. Nonetheless, the low incidence of HSCC makes it difficult to conduct large clinical trials, which underscores the importance of our study. The development of predictive models to guide clinical decision making is critical to provide optimal treatment strategies for patients with HSCC. In future research, we will further validate the performance of our model using external data. It is necessary to consider conducting multi-center clinical studies in the Chinese population to further validate the clinical utility of the nomogram and provide more convincing evidence.

## 5. Conclusions

Our study established a nomogram of postoperative HSCC based on lymph node parameters, which could significantly distinguish patients with different risks and could predict their prognosis. This nomogram demonstrated good performance and could serve as a practical tool for clinicians to select chemotherapy candidates for HSCC patients.

## Figures and Tables

**Table 1:** General Characteristics.

Characteristics	Entire Population		Training Cohort		Validation Cohort		*p*
	n = 404	%	n = 282	%	n = 122	%	
**Year of diagnosis**							0.765
2004–2011	190	47.03	134	47.52	56	45.90	
2011–2019	214	52.97	148	52.48	66	54.10	
**Age at diagnosis (year)**							0.443
Median (range)	62	26–87	62	30–87	62	26–85	
**Origin**							0.322
Non-Spanish–Hispanic–Latino	369	91.34	255	90.43	114	93.44	
Spanish–Hispanic–Latino	35	8.66	27	9.57	8	6.56	
**Race**							0.611
White	313	77.48	218	77.3	95	77.87	
Black	57	14.11	38	13.48	19	15.57	
Other	34	8.42	26	9.22	8	6.56	
**Sex**							0.962
Male	335	82.92	234	82.98	101	82.79	
Female	69	17.08	48	17.02	21	17.21	
**Married**							0.467
Married	181	44.80	123	43.62	58	47.54	
No/Unknown	223	55.20	159	56.38	64	52.46	
**Primary site**							0.328
Pyriform sinus	236	58.42	165	58.51	71	58.20	
Other sites	68	16.83	43	15.25	25	20.49	
Hypopharynx, NOS	100	24.75	74	26.24	26	21.31	
**Pathological grade**							0.366
I	11	2.72	5	1.77	6	4.92	
II	195	48.27	139	49.29	56	45.90	
III	169	41.83	120	42.55	49	40.16	
IV	6	1.49	4	1.42	2	1.64	
Unknown	23	5.69	14	4.96	9	7.38	
**SEER stage**							0.652
Localized	30	7.43	20	7.09	10	8.20	
Regional	259	64.11	178	63.12	81	66.39	
Metastasis	115	28.47	84	29.79	31	25.41	
**AJCC TNM stage**							0.595
I	8	1.98	4	1.42	4	3.28	
II	19	4.70	12	4.26	7	5.74	
III	35	8.66	27	9.57	8	6.56	
IVA	296	73.27	207	73.4	89	72.95	
IVB	46	11.39	32	11.35	14	11.48	
**T stage**							0.538
T1	26	6.44	17	6.03	9	7.38	
T2	76	18.81	54	19.15	22	18.03	
T3	61	15.10	47	16.67	14	11.48	
T4a	219	54.21	149	52.84	70	57.38	
T4b	22	5.45	15	5.32	7	5.74	
**N stage**							0.585
N0	76	18.81	54	19.15	22	18.03	
N1	70	17.33	44	15.6	26	21.31	
N2	230	56.93	164	58.16	66	54.10	
N3	28	6.93	20	7.09	8	6.56	
**Tumor size (cm)**							0.849
≤2	51	12.62	35	12.41	16	13.11	
2–4	146	36.14	101	35.82	45	36.89	
>4	192	47.52	134	47.52	58	47.54	
Unknown	15	3.71	12	4.26	3	2.46	
**ELN**							0.340
Median (range)	43	1–90	44	1–90	42	1–90	
**PLNN**							0.494
Median (range)	2	0–34	3	0–34	2	0–24	
**LNR**							0.785
Median (range)	0.06	0–1	0.06	0–1	0.05	0–1	
**Surgery**		0.00		0		0.00	0.289
Pharyngectomy	86	21.29	55	19.5	31	25.41	
Pharyngectomy with laryngectomy or removal of contiguous bone tissue	271	67.08	191	67.73	80	65.57	
Radical pharyngectomy	47	11.63	36	12.77	11	9.02	
**Radiotherapy**						0.00	0.104
No	101	25.00	64	22.7	37	30.33	
Yes	303	75.00	218	77.3	85	69.67	
**Chemotherapy**						0.00	0.141
No	206	50.99	137	48.58	69	56.56	
Yes	198	49.01	145	51.42	53	43.44	

Abbreviation: SEER, Surveillance, Epidemiology, and End Results; AJCC, American Joint Committee on Cancer; ELN, examined lymph node; PLNN, positive lymph node number; LNR, lymph node ratio.

**Table 2:** Univariate and multivariate survival analysis on OS for patients with HSCC after surgery.

Characteristics	Univariate	*p*	Multivariate	*p*
	HR (95% CI)		HR (95% CI)	
**Year of diagnosis**				
2004–2011	1			
2011–2019	1.009 (0.738–1.381)	0.953		
**Age at diagnosis (year)**				
≤71	1		1	
>71	1.736 (1.203–2.506)	0.003	2.011 (1.356–2.983)	0.001
**Origin**				
Non-Spanish–Hispanic–Latino			1	
Spanish–Hispanic–Latino	0.870 (0.521–1.454)	0.595	0.497 (0.287–0.861)	0.013
**Race**				
White	1			
Black	0.856 (0.53–1.381)	0.523		
Other	0.910 (0.525–1.576)	0.735		
**Sex**				
Male				
Female	1.056 (0.717–1.556)	0.781		
**Married**				
Married	1			
No/Unknown	1.294 (0.961–1.743)	0.090		
**Primary site**				
Pyriform sinus	1		1	
Other sites	0.993 (0.656–1.502)	0.973	0.821 (0.533–1.265)	0.372
Hypopharynx, NOS	0.611 (0.419–0.892)	0.011	0.522 (0.350–0.778)	0.001
**Pathological grade**				
I	1			
II	0.763 (0.279–2.086)	0.597		
III	0.870 (0.317–2.383)	0.786		
IV	2.087 (0.462–9.424)	0.339		
Unknown	0.870 (0.276–2.747)	0.812		
**T stage**				
T1	1		1	
T2	3.273 (1.292–8.289)	0.012	4.123 (1.589–10.698)	0.004
T3	2.871 (1.105–7.462)	0.030	5.024 (1.861–13.562)	0.001
T4	3.554 (1.448–8.724)	0.006	5.733 (2.264–14.518)	0.001
**N stage**				
N0	1			
N1	1.453 (0.863–2.447)	0.160		
N2	1.960 (1.267–3.033)	0.002		
N3	2.821 (1.343–5.927)	0.006		
**Tumor size**				
≤2	1			
2–4	2.017 (1.170–3.476)	0.012		
>4	2.168 (1.270–3.703)	0.005		
Unknown	2.552 (1.124–5.797)	0.025		
**ELN**				
1–36	1		1	
37–72	1.253 (0.91–1.727)	0.167	1.339 (0.935–1.917)	0.111
73–90	1.815 (1.164–2.832)	0.009	1.947 (1.153–3.287)	0.013
**PLNN**				
0–1	1			
2–6	1.629 (1.152–2.303)	0.006		
7–34	2.998 (2.006–4.479)	0.000		
**LNR**				
0–0.03	1		1	
0.03–0.23	1.797 (1.279–2.526)	0.001	2.721 (1.492–4.963)	0.001
0.23–1.00	2.554 (1.633–3.996)	<0.001	3.776 (1.713–8.324)	0.001
**Surgery**				
Pharyngectomy	1			
Pharyngectomy with laryngectomy or removal of contiguous bone tissue	1.072 (0.740–1.554)	0.713		
Radical pharyngectomy	1.293 (0.794–2.104)	0.302		
**Radiotherapy**				
No	1		1	
Yes	0.525 (0.379–0.728)	<0.001	0.294 (0.205–0.423)	<0.001

Abbreviation: ELN, examined lymph node; PLNN, positive lymph node number; LNR, lymph node ratio. OS, overall survival; HSCC, Hypopharyngeal squamous cell carcinoma; NOS, not otherwise specified; HR, hazard ratio; CI, confidence interval.

## Data Availability

All data can be retrieved from the public SEER database.

## Supplementary Materials

The following supporting information can be downloaded at: https://www.mdpi.com/article/10.3390/jcm11195801/s1, Figure S1: Flow chart. Abbreviation: NOS, not otherwise specified; NA, not avaliable; ELN, examined lymph nodes; LNN, lymph nodes number; Figure S2: The optimal cutoff values for age (A), ELN (B), PLNN (C), and LNR (D). Abbreviation: ELN, examined lymph node; PLNN, positive lymph node number; LNR, lymph node ratio.
