# Supplementary material for: Prognostic Nomogram for Postoperative Hypopharyngeal Squamous Cell Carcinoma to Assist Decision Making for Adjuvant Chemotherapy
Source: J Clin Med. 2022 Sep 30;11(19):5801. doi: 10.3390/jcm11195801 (PMC9573651; doi:10.3390/jcm11195801)
Supplement: Supplementary file 1 [file jcm-11-05801-s001.zip › jcm-1903346-supplementary.pdf]

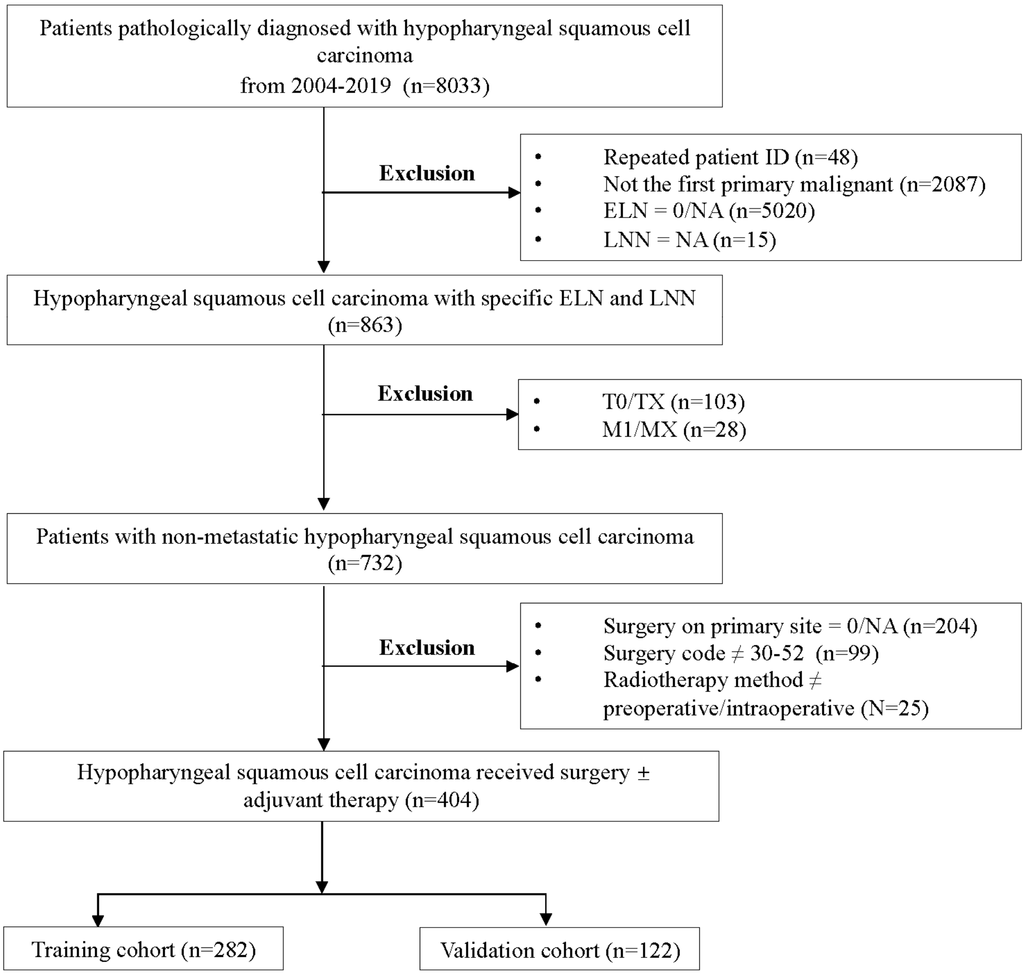

Supplementary Figure S1. Flow chart. Abbreviation: NOS, not otherwise specified; NA, not avaliable; ELN, examined lymph nodes; LNN, lymph nodes number.

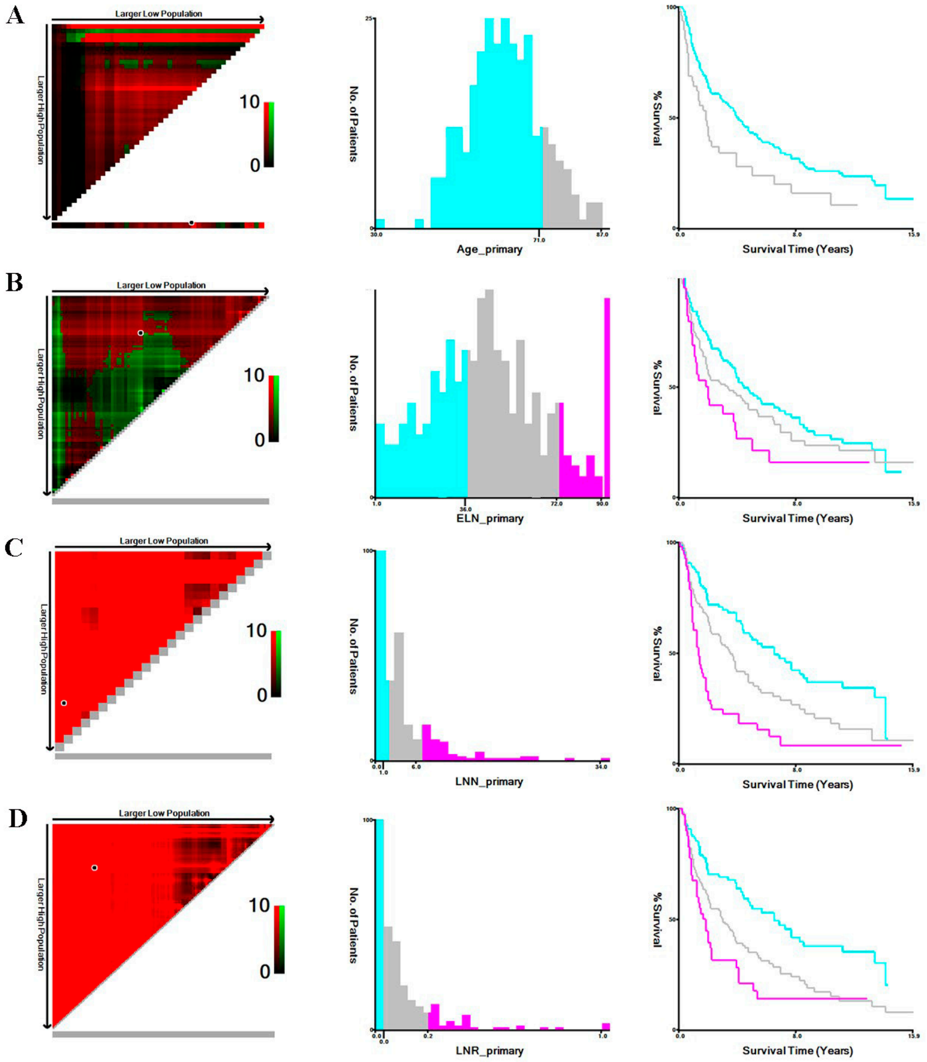

Supplementary Figure S2. The optimal cutoff values for age (A), ELN (B), PLNN (C), and LNR (D). Abbreviation: ELN, examined lymph node; PLNN, positive lymph node number; LNR, lymph node ratio.
